# Sparse sampling: theory, methods and an application in neuroscience

**DOI:** 10.1007/s00422-014-0639-x

**Published:** 2014-12-02

**Authors:** Jon Oñativia, Pier Luigi Dragotti

**Affiliations:** Communications and Signal Processing Group, Department of Electrical and Electronic Engineering, Imperial College London, London, SW7 2AZ UK

**Keywords:** Sampling theory, FRI, Spike train inference, Calcium transient

## Abstract

The current methods used to convert analogue signals into discrete-time sequences have been deeply influenced by the classical Shannon–Whittaker–Kotelnikov sampling theorem. This approach restricts the class of signals that can be sampled and perfectly reconstructed to bandlimited signals. During the last few years, a new framework has emerged that overcomes these limitations and extends sampling theory to a broader class of signals named signals with finite rate of innovation (FRI). Instead of characterising a signal by its frequency content, FRI theory describes it in terms of the innovation parameters per unit of time. Bandlimited signals are thus a subset of this more general definition. In this paper, we provide an overview of this new framework and present the tools required to apply this theory in neuroscience. Specifically, we show how to monitor and infer the spiking activity of individual neurons from two-photon imaging of calcium signals. In this scenario, the problem is reduced to reconstructing a stream of decaying exponentials.

## Introduction

The world is analogue, but computation is digital. The process that bridges this gap is known as the sampling process and has been instrumental to the digital revolution of the past 60 years. Without the sampling process, we could not convert real-life signals in digital form, and without digital samples, we could not use computers for digital computation. The sampling process is also ubiquitous in that it is present in any mobile phone or digital camera but also in sophisticated medical devices like MRI or ultrasound machines, in sensor networks and in digital microscopes just to name a few examples.

Over the last six decades, our understanding of the conversion of continuous-time signal in discrete form has been heavily influenced by the Shannon–Whittaker–Kotelnikov sampling theorem (Shannon 1949; Whittaker 1929; Kotelnikov 1933; Unser 2000) which showed that the sampling and perfect reconstruction of signals are possible when the Fourier bandwidth or spectrum of the signal is finite. In this case, the signal is said to be bandlimited and must be sampled at a rate (Nyquist rate) at least twice its maximum nonzero frequency in order to reconstruct it without error.

**Fig. 1:**
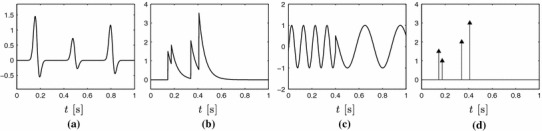
Examples of signals with FRI. When the shape of the pulse is known, the signal depends only on the amplitude and location of such pulses

We are so used to this approach that we tend to forget that it comes with many strings attached. First of all, there are no natural phenomena that are exactly bandlimited (Slepian 1976). Moreover, we tend to forget that the Shannon sampling theorem provides sufficient but not necessary conditions for perfect reconstruction. In other words, this theorem does not claim that it is not possible to sample and reconstruct non-bandlimited signals. It is therefore incorrect to assume that the bandwidth of a signal is related to its information content. Consider for instance the function shown in Fig. 1a. This is a stream of short pulses and appears in many applications including bio-imaging, seismic signals and spread-spectrum communication. If the pulse shape is known a priori, the signal is completely determined by the amplitude and location of such pulses. If there are at most $K$ pulses in a unit interval, then the signal is completely specified by the knowledge of these $2K$ parameters per unit of time. Assume now that the duration of the pulses is reduced but that the average number of pulses per unit interval stays the same. Clearly, the information content of the signal is not changing (still $2K$ parameters per unit of time); however, its bandwidth is increasing (bandwidth increases when the support of a function decreases).

**Fig. 2:**
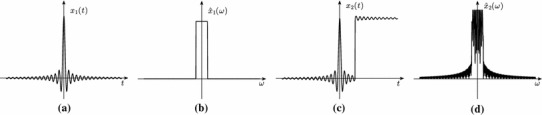
Nyquist rate versus information rate. The signal $x_1(t)$ depicted in part **a** is bandlimited as shown in part **b**. The sum of $x_1(t)$ with a step function lead to a signal $x_2(t)$ with infinite bandwidth as shown in part **c** and **d**

Consider, as second example, the signal shown in Fig. 2c. This is given by the sum of a bandlimited signal with a step function. Clearly, the step function has only two degrees of freedom: the discontinuity location and its amplitude. So, its information content is finite. The bandlimited function has a finite number of degrees of freedom per unit of time since it is fully determined by its samples at points spaced by the sampling period (given by the inverse of the Nyquist rate). We thus say that they both have a finite rate of innovation. However, the combination of these two functions leads to a signal with infinite bandwidth (see Fig. 2d). Now, if we were to relate the information content of the signal to its bandwidth, we would conclude incorrectly that this signal has an infinite rate of information since it requires an infinite sampling rate for perfect reconstruction. Therefore, bandwidth and information content are not always synonyms.

A first attempt to reconcile these two notions: sampling rate and information content was made in Vetterli et al. (2002). Here, they introduced a new class of signals called signals with finite rate of innovation (FRI) which includes both bandlimited signals and the non-bandlimited functions discussed so far. They went on showing that classes of FRI signals can be sampled and perfectly reconstructed using an appropriate acquisition device. These results have then be extended to include more classes of acquisition devices (Dragotti et al. 2007; Seelamantula and Unser 2008; Asl et al. 2010; Tur et al. 2011; Urigüen et al. 2013) and more classes of signals (Maravić and Vetterli 2005; Berent et al. 2010; Chen et al. 2012). FRI sampling theory has also had impact in various applications (Baboulaz and Dragotti 2009; Poh and Marziliano 2010; Tur et al. 2011; Kandaswamy et al. 2013) and here we focus on an application in neuroscience.

The paper is organised as follows. In the next section, we define FRI signals and give some examples. Section 3 presents the framework for sampling and reconstructing some classes of FRI signals. Specifically, we show how to sample and perfectly reconstruct a stream of Diracs and what are the conditions that the acquisition device has to satisfy. We also extend this framework to the case of streams of decaying exponentials and present some denoising strategies. Section 4 presents an algorithm to reconstruct streaming signals where there is no clear separation between consecutive bursts of spikes. Section 5 describes an application of this theory to monitor neural activity from two-photon calcium images. Finally, conclusions are drawn in Sect. 6.

### Notations

For $f(t) \in \varvec{L^2}(\mathbb {R})$, where $\varvec{L^2}(\mathbb {R})$ is the Hilbert space of finite-energy functions, the Fourier transform of $f(t)$ is denoted by $\hat{f}(\omega )$ and is given by $\hat{f}(\omega ) = \mathcal {F}\lbrace f(t)\rbrace = \int _{-\infty }^{+\infty } f(t) e^{-i \omega t} \hbox {d}t$. If $f(t)$ is complex-valued, $f^*(t)$ denotes its complex conjugate. The Hermitian inner product is $\left\langle f\,,\,g\right\rangle = \int _{-\infty }^{+\infty } f(t) g^*(t) \hbox {d}t$. The indicator function is denoted by $\varvec{1}_A(t)$ and is given by $\varvec{1}_A(t)=1$ if $t \in A$, and $\varvec{1}_A(t)=0$ if $t \notin A$. $\delta _{i,j}$ denotes the Kronecker delta, which is defined as $\delta _{i,j} = 1$ if $i=j$ and 0 otherwise. $\lfloor {\cdot }\rfloor$ and $\lceil {\cdot }\rceil$ denote the floor and ceil functions.

## Finite rate of innovation signals

Classical sampling theorems state that any bandlimited function $x(t)$ such that $\hat{x}(\omega ) = 0, \forall \omega > \omega _{\max }$, can be perfectly recovered from its samples $x_n = x(t)|_{t=nT}$ if the sampling rate $2 \pi / T$ is greater than or equal to twice the highest frequency component of $x(t)$, that is, $2\pi /T\ge \omega _{\max }$. Moreover, the original signal can be perfectly reconstructed as follows:

1 $$\begin{aligned} x(t) = \sum _{n=-\infty }^{\infty } x_n \, \text {sinc}(t/T - n), \end{aligned}$$

where $\text {sinc}(t) = \sin (\pi t) / \pi t$. If $x(t)$ is not bandlimited, sampling with an ideal lowpass filter ($h(t) = \text {sinc}(t/T)$) and reconstruction applying (1) provides a lowpass approximation of $x(t)$. This is the best approximation in the least square sense of $x(t)$ in the space spanned by $\lbrace \text {sinc}(t/T - n) \rbrace _{n \in \mathbb {Z}}$ (Unser 2000). However, it is an approximation, and perfect reconstruction of the original signal is not achieved. We also note that signals defined as in (1) are completely specified by the knowledge of a new parameter $x_n$ every $T$ seconds.

Based on this observation, consider now a new class of signals that extend the one in (1) (Vetterli et al. 2002):

2 $$\begin{aligned} x(t)=\sum _{k \in \mathbb {Z}} \sum _{r=0}^{R} \, a_{r,k} \, g_r(t-t_k), \end{aligned}$$

where $\lbrace g_r(t) \rbrace _{r=0}^{R}$ is a set of known functions. We note that, since $g_r(t)$ are known, signals in (2) are uniquely determined by the set of parameters $a_{r,k}$ and $t_k$. Introducing a counting function $C_x(t_a,t_b)$ that counts the number of degrees of freedom in $x(t)$ over the interval $\left[ t_a, t_b\right]$, we define the rate of innovation $\rho$ as follows (Vetterli et al. 2002; Dragotti et al. 2007; Blu et al. 2008; Urigüen et al. 2013):

3 $$\begin{aligned} \rho = \lim _{\tau \rightarrow \infty } \frac{1}{\tau } \, C_x \left( -\frac{\tau }{2},\frac{\tau }{2}\right) \end{aligned}$$

and signals with a finite $\rho$ are called signals with a finite rate of innovation (FRI).

It is of interest to note that bandlimited signals fall under this definition. Therefore, one possible interpretation is that it is possible to sample them because they have a finite rate of innovation (rather than because they are bandlimited). Examples of FRI signals which are not bandlimited and which are of interest to us include

- Stream of pulses: $x(t)=\sum _k a_k \, p(t-t_k)$. For instance, stream of decaying exponentials:
  4 $$\begin{aligned} x(t)=\sum _{k} a_k \, e^{-(t-t_k) / \tau } \, \varvec{1}_{t \ge t_k}, \end{aligned}$$
  which are a good fit for calcium transient signals induced by neural activity in two-photon calcium imaging. Figure 1a, b are examples of such signals.
- Piecewise sinusoidal signals (see Fig. 1c):
  5 $$\begin{aligned} x(t) = \sum _k \sum _r a_{k,r} \, e^{i (\omega _{k,r}t + \phi _{k,r})} \, \varvec{1}_{[t_k,t_{k+1})}(t). \end{aligned}$$
- Stream of Diracs (see Fig. 1d):
  6 $$\begin{aligned} x(t) = \sum _k a_k \, \delta (t-t_k). \end{aligned}$$

## Sampling scheme

**Fig. 3:**
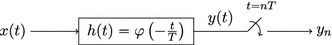
Acquisition process

Consider the typical acquisition process as shown in Fig. 3. This is usually modelled as a filtering stage followed by a sampling stage. The filter accounts for the modifications that the analogue signal $x(t)$ experiences before being sampled. It may model an anti-aliasing filter or it might be due to the distortion introduced by the acquisition device, for example, in the case of a digital camera the distortion due to the lens. Filtering signal $x(t)$ with $h(t)=\varphi (-t/T)$ and retrieving samples at instants of time $t=n\,T$ is equivalent to computing the inner product between $x(t)$ and $\varphi (t/T-n)$. Specifically, the filtered signal is given by

7 $$\begin{aligned} \begin{aligned} y(t)&= x(t) * h(t) \\&= \int _{-\infty }^{+\infty } x(\tau ) h(t-\tau ) \hbox {d}\tau \\&= \int _{-\infty }^{+\infty } x(\tau ) \varphi \left( -\frac{t-\tau }{T}\right) \hbox {d}\tau . \end{aligned} \end{aligned}$$

Moreover, sampling $y(t)$ at regular intervals of time $t=n\,T$ leads to

8 $$\begin{aligned} \begin{aligned} y_n&= y(t) |_{t=nT}\\&= \int _{-\infty }^{+\infty } x(\tau ) \varphi (\tfrac{\tau }{T} - n) \hbox {d}\tau \\&= \left\langle x(t)\,,\,\varphi (\tfrac{t}{T} - n)\right\rangle . \end{aligned} \end{aligned}$$

The function $\varphi (t)$ is called the sampling kernel. In order to guarantee perfect reconstruction of the signal $x(t)$, the sampling kernel and the input signal have to satisfy some conditions. The literature presents a variety of kernels that can be used to achieve perfect reconstruction of FRI signals. Here, we will focus on exponential reproducing kernels since they offer the best flexibility and resilience to noise.

- *Exponential reproducing property*: Any function $\varphi (t)$ that together with its shifted versions can reproduce exponential functions of the form $e^{\alpha _m t}$ with $\alpha _m \in \mathbb {C}$ and $m = 0,1,\ldots ,P$:
  9 $$\begin{aligned} \sum _{n\in \mathbb {Z}} c_{m,n} \,\varphi (t-n) = e^{\alpha _m t}, \quad m = 0,1,\ldots ,P. \end{aligned}$$

**Fig. 4:**
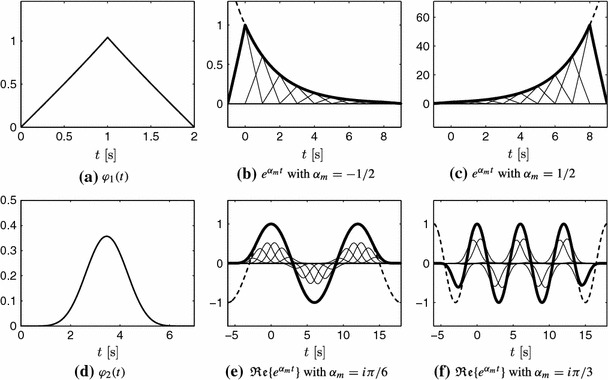
Two different kernels that reproduce exactly different exponentials. The *first row* illustrates the reproduction of two real exponential functions with the kernel $\varphi _1(t)$, shown in **a**. The *second row* illustrates the reproduction of two complex exponentials with the kernel $\varphi _2(t)$, shown in **d**. In **e** and **f**, only the real part of the exponentials is shown ($e^{\alpha _m t} = e^{i \omega _m t} = \cos (\omega _m t) + i \sin (\omega _m t)$). The *thin lines* represent the shifted and weighted kernels, the *thick line* represents their sum and the *dashed line* the true exponential. Note that both kernels are of compact support. The summation in (9) is truncated which leads to the border effects in **b**, **c**, **e** and **f**

The exponential reproduction property is illustrated in Fig. 4 for two different kernels that reproduce different exponentials. In both cases, the kernels are of compact support. The advantage of such kernels is that the summation in (9) can be truncated and still have a region in time where the exponential functions are perfectly reproduced. In general, the exponentials $e^{\alpha _m t}$ are perfectly reproduced when the summation is computed for $n \in \mathbb {Z}$. Let $t \in [0, L)$ be the support of $\varphi (t)$, that is, $\varphi (t) = 0$ for $t \notin [0, L)$. If the summation is truncated to $n = n_0, \ldots , n_f$, it follows that the perfect reproduction of the exponential functions holds for $t \in [n_0-1+L, n_f+1)$.

### Exponential reproducing kernels

For the sake of clarity, in what follows, we restrict the analysis to the case where the parameter $\alpha _m$ in (9) is purely imaginary, that is $\alpha _m = i \omega _m$ for $m = 0, 1, \ldots , P$, where $\omega _m\in \mathbb {R}$. This analysis can easily be extended to the more general case where $\alpha _m$ has nonzero real and imaginary parts, or is purely real.

A function $\varphi (t)$ together with a linear combination of its shifted versions reproduces the exponentials $\lbrace e^{i \omega _m t}\rbrace _{m=0}^P$ as in (9) if and only if it satisfies the generalised Strang-Fix conditions:

10 $$\begin{aligned} \hat{\varphi }(\omega _m) \ne 0 \quad \text {and} \quad \hat{\varphi }(\omega _m + 2 \pi l) = 0, \end{aligned}$$

where $m=0,1,\ldots ,P$, $l\in \mathbb {Z} \, \setminus \, \lbrace 0 \rbrace$ and $\hat{\varphi }(\omega )$ is the Fourier transform of $\varphi (t)$ (Strang and Fix 1971; Unser and Blu 2005; Urigüen et al. 2013). A family of functions that satisfy these conditions are the exponential B-splines, also named E-splines. These functions are constructed through the convolution of elementary zero order E-splines, where each elementary function reproduces a particular exponential $e^{i \omega _m t}$. The Fourier transform of a zero order E-spline that reproduces the exponential $e^{\alpha t}$ is given by

11 $$\begin{aligned} \hat{\beta }_\alpha (\omega ) = \frac{1-e^{\alpha -i\,\omega }}{i\,\omega - \alpha }. \end{aligned}$$

**Fig. 5:**
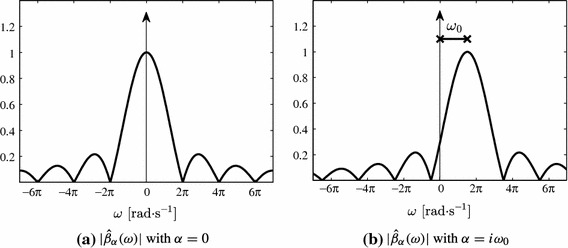
Absolute value of the Fourier transform of zero order E-splines given by (11). If the parameter $\alpha$ is equal to zero, the E-spline corresponds to the sinc function. For $\alpha =i\omega _0$ purely imaginary, the Fourier transform of the E-spline is a shifted version of the sinc function

Figure 5 illustrates the Fourier transform of zero order E-splines for two different values of the parameter $\alpha$.

The corresponding E-spline that reproduces the set of exponentials $\lbrace e^{\alpha _m t} \rbrace _{m=0}^{P}$ is obtained as follows

12 $$\begin{aligned} \beta _{\varvec{\alpha }} (t) = \left( \beta _{\alpha _0} * \beta _{\alpha _1} * \cdots * \beta _{\alpha _P} \right) (t), \end{aligned}$$

where $\varvec{\alpha } = \left( \alpha _0, \alpha _1, \ldots , \alpha _P \right)$. Thus, the Fourier transform of $\beta _{\varvec{\alpha }} (t)$ is given by

13 $$\begin{aligned} \hat{\beta }_{\varvec{\alpha }} (\omega ) = \prod _{m=0}^{P} \left( \frac{1-e^{\alpha _m-i\,\omega }}{i\,\omega - \alpha _m} \right) . \end{aligned}$$

E-splines have compact support $P+1$ and have $P-1$ continuous derivatives. It can be shown that any function that reproduces the set of exponentials $\lbrace e^{\alpha _m t} \rbrace _{m=0}^{P}$ can be expressed as the convolution of another function $\gamma (t)$ with the corresponding E-spline that reproduces these exponentials, that is, $\varphi (t) = \gamma (t) * \beta _{\varvec{\alpha }} (t)$ and $\gamma (t)$ satisfies $\int _{-\infty }^{+\infty } e^{-\alpha _m t} \gamma (t) \hbox {d}t \ne 0$ for all $\alpha _m$(Unser and Blu 2005; Delgado-Gonzalo et al. 2012). It is also true that if $\varphi (t)$ reproduces a set of exponentials, this property is preserved through convolution. Let

14 $$\begin{aligned} \psi (t)=\varphi (t)*\rho (t), \end{aligned}$$

for $\rho (t)$ such that $\int _{-\infty }^{+\infty } e^{-\alpha _m t} \rho (t) \hbox {d}t \ne 0$. The function $\psi (t)$ also reproduces the same set of exponentials. This is easy to verify since $\psi (t)$ also satisfies the Strang-Fix conditions.

#### Sampling with an exponential reproducing kernel

The choice of purely imaginary parameters $\alpha _m = i \omega _m$ leads to an important family of sampling kernels. These design parameters directly determine the information of the input analogue signal $x(t)$ that we acquire and allow us to perfectly reconstruct the input signal from the discrete samples $y_n$ for some classes of signals. Specifically, the different $\omega _m$ correspond to the frequencies of the Fourier transform of $x(t)$ that we are able to retrieve from the only knowledge of samples $y_n$. It can be shown that if parameters $\alpha _m$ are real or appear in complex conjugate pairs, the corresponding E-spline is real. We thus impose that for all $\alpha _m$ that are nonzero, their complex conjugates are also present in $\varvec{\alpha }$. If parameters $\alpha _m = i \omega _m$ in vector $\varvec{\alpha }$ are sorted in increasing order of $\omega _m$, we have that $\alpha _m^* = \alpha _{P-m}$.

Let us assume that function $x(t)$ is localised in time and thus only $N$ samples $y_n$ are nonzero. Let $\left( s_m\right) _{m=0}^P$ be the sequence obtained by linearly combining samples $y_n$ with the coefficients $c_{m,n}$ from (9), that is, $s_m = \sum _{n=1}^{N} c_{m,n} \, y_n$. We have that

15 $$\begin{aligned} \begin{aligned} s_m&\overset{(a)}{=} \sum _{n=1}^{N} c_{m,n} \, \left\langle x(t)\,,\,\varphi (t/T-n)\right\rangle \\&\overset{(b)}{=} \int _{-\infty }^{+\infty } x(t) \sum _{n=1}^{N} c_{m,n} \, \varphi (t/T-n) \hbox {d}t\\&\overset{(c)}{=} \int _{-\infty }^{+\infty } x(t) \, e^{i \omega _m t / T} \hbox {d}t = \hat{x} (-\omega _m/T), \end{aligned} \end{aligned}$$

where $(a)$ follows from (8), $(b)$ from the linearity of the inner product and $(c)$ from the exponential reproduction property. The quantity $s_m$ therefore corresponds to the Fourier transform of $x(t)$ evaluated at $\omega = -\omega _m / T$. Since we have imposed $-\omega _m = \omega _{P-m}$, we also have that $s_{P-m} = \hat{x} (\omega _m/T)$.

#### Computation of $c_{m,n}$ coefficients

We have established the properties that a function $\varphi (t)$ has to satisfy in order to reproduce exponentials, which are given by the Strang-Fix conditions. Moreover, we have seen the importance of the E-splines since they allow us to obtain samples of the Fourier transform of the input signal. We now show how to obtain the coefficients $c_{m,n}$ in (9) required to reproduce the exponential functions $\lbrace e^{i \omega _m t} \rbrace _{m=0}^P$, and that are used to obtain the sequence $s_m$ in (15). These coefficients are given by

16 $$\begin{aligned} c_{m,n} = \int _{-\infty }^{\infty } e^{i \omega _m t} \tilde{\varphi } (t-n) \hbox {d}t, \end{aligned}$$

where $\tilde{\varphi }(t)$ is chosen to form with $\varphi (t)$ a quasibiorthonormal set (Dragotti et al. 2007). This includes the particular case where $\tilde{\varphi }(t)$ is the dual of $\varphi (t)$, that is, $\left\langle \tilde{\varphi }(t-n), \varphi (t-m) \right\rangle = \delta _{n,m}$. The introduction of $\tilde{\varphi }(t)$ is a technicality that is needed in order to show where the coefficients $c_{m,n}$ come from, but we do not need to work with this function. From (16), we can express $c_{m,n}$ in terms of $c_{m,0}$ by applying a change of variable $t'=t-n$:

17 $$\begin{aligned} \begin{aligned} c_{m,n}&= e^{i \omega _m n} \int _{-\infty }^{\infty } e^{i \omega _m t} \, \tilde{\varphi } (t) \hbox {d}t\\&= e^{i \omega _m n} \, c_{m,0}. \end{aligned} \end{aligned}$$

If we plug this expression in (9), we can derive an expression to compute $c_{m,0}$ for each $m=0,\ldots ,P$:

18 $$\begin{aligned} c_{m,0} = \left( \sum _{\,n \in \mathbb {Z}} e^{-i \omega _m (t-n)} \, \varphi (t-n)\right) ^{-1}, \, m = 0,1,\ldots ,P, \end{aligned}$$

which is valid for any value of $t$. Let $\psi (t) : = e^{-i \omega _m t} \varphi (t)$, we have that

19 $$\begin{aligned} \begin{aligned} \sum _{n \in \mathbb {Z}} e^{-i \omega _m (t-n)} \, \varphi (t-n)&= \sum _{n \in \mathbb {Z}} \psi (t-n)\\&\overset{(a)}{=} \sum _{k \in \mathbb {Z}} \hat{\psi } (2 \pi k) \, e^{i 2 \pi k t}\\&\overset{(b)}{=} \sum _{k \in \mathbb {Z}} \hat{\varphi } (\omega _m + 2 \pi k) \, e^{i 2 \pi k t}, \end{aligned} \end{aligned}$$

where $(a)$ follows from the Possion summation formula^1^ and $(b)$ from the fact that the Fourier transform of $\psi (t)$ is equal to the Fourier transform of $\varphi (t)$ shifted by $\omega _m$. Since $\hat{\varphi }(\omega )$ satisfies the Strang-Fix conditions, from (18) and (19) it follows that

20 $$\begin{aligned} c_{m,0} = \left[ \hat{\varphi }(\omega _m) \right] ^{-1}. \end{aligned}$$

**Fig. 6:**
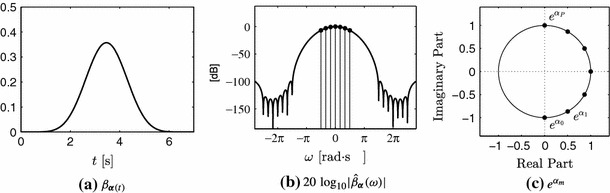
E-spline of order $P=6$ and $\varvec{\alpha } = \left[ -i\pi /2, -i\pi /3,-i\pi /6, 0, i\pi /6, i\pi /3, i\pi /2\right]$. Note that the support in time is equal to $P+1$ and quickly decays to zero. This E-spline reproduces the exponentials in Fig. 4 among others. Parameters $\alpha _m$ are purely imaginary or equal to zero; purely imaginary $\alpha _m$ appear in complex conjugate pairs. **a** illustrates the shape of the E-spline in time and **b** in frequency (expressed in dB). The *dots* in **b** represent the locations at which the Fourier transform of $\varphi (t)$ is sampled in order to compute the $c_{m,0}$ coefficients as in (20). **c** is a representation of the complex values $e^{\alpha _m}$

The dots in Fig. 6b illustrate the values $\hat{\varphi }(\omega _m)$ that are used in the computation of the different $c_{m,0}$ for an E-spline with $P=6$. Note that the generalised Strang-Fix conditions (10) impose some constraints on the choice of $\omega _m$ since we have to guarantee that $\hat{\varphi }(\omega _m) \ne 0$. From (11) and Fig. 5, it is clear that each $\omega _m$ introduces zeros at locations $\omega _m + 2 \pi l$, where $l \in \mathbb {Z} \setminus \{0\}$, we thus have to guarantee that for all pairs of distinct $m,n$ we have $\omega _m - \omega _n \ne 2 \pi l$. In Fig. 6b, it can be appreciated that $\hat{\varphi }(\omega )$ is nonzero for all $\omega =\omega _m$, and that the locations $\omega _m + 2 \pi$ and $\omega _m - 2 \pi$ are zero since the curve in dB tends to $-\infty$.

From (20) and (17), we can compute the $c_{m,n}$ coefficients for our choice of $\left( \alpha _m\right) _{m=0}^{P}$ and any value of $n \in \mathbb {Z}$. By combining these coefficients with $\lbrace \varphi (t-n)\rbrace _{n\in \mathbb {Z}}$, the exponentials $\lbrace e^{\alpha _m t} \rbrace _{m=0}^P$ are perfectly reproduced as shown in Fig. 4.

#### Approximate reproduction of exponentials

The generalised Strang-Fix conditions (10) impose restrictive constraints on the sampling kernel. This becomes a problem when we do not have control or flexibility over the design of the acquisition device. Recent publications (Urigüen et al. 2013; Dragotti et al. 2013) show that these conditions can be relaxed and still have a very accurate exponential reproduction, which is the property we require in order to reconstruct the analogue input signal. The first part of the Strang-Fix conditions, that is $\hat{\varphi }(\omega _m) \ne 0$, is easy to achieve, but the second part is harder to guarantee when we do not have control over the sampling device.

If the sampling kernel does not satisfy the generalised Strang-Fix conditions, the exponential reproduction property (9) cannot be satisfied exactly. We thus have to find the coefficients $c_{m,n}$ that better approximate the different exponentials $e^{i \omega _mt}$:

21 $$\begin{aligned} \sum _{n \in \mathbb {Z}} c_{m,n} \, \varphi (t-n) \simeq e^{i \omega _m t}. \end{aligned}$$

There are various options to compute these coefficients, but a good and stable approximation is obtained with the *constant least squares* approach (Urigüen et al. 2013). If the Fourier transform of the sampling kernel is sufficiently small at $\omega = \omega _m + 2 \pi l$, $l \ne 0$, the $c_{m,n}$ coefficients are given by

22 $$\begin{aligned} c_{m,n} = \hat{\varphi } (\omega _m) \, e^{i \omega _m n}. \end{aligned}$$

**Fig. 7:**
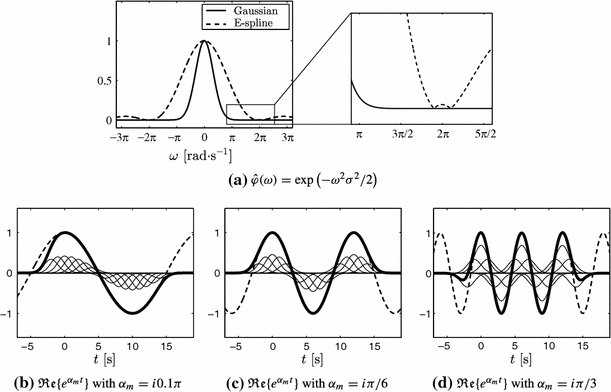
Gaussian sampling filter with $\sigma =1$ and approximate exponential reproduction. **a** Fourier transform of Gaussian function compared to a first order E-spline that reproduces frequencies $-0.1\pi$ and $0.1\pi$. The zoom-in box shows the region where the E-spline is zero ($\omega =\omega _m + 2 \pi l$); the Gaussian function has a negligible amplitude. **b**–**d** are examples of exponential reproduction with the Gaussian filter. As in Fig. 4, the *thin lines* represent the shifted and weighted versions of $\varphi (t)$ (which in this case is the Gaussian function), the *thick line* their sum, and the *dashed line* the true exponentials

Gaussian filters are good candidates for this approach since they are smooth and the shape in time is very similar to the E-splines (see Fig. 7a). The Fourier transform of such filters is given by

23 $$\begin{aligned} \varphi (t) = \frac{1}{\sqrt{2 \pi \sigma ^2}} \, e^{-t^2 / 2 \sigma ^2} \, \overset{\mathcal {F}}{\longrightarrow } \, \hat{\varphi } (\omega ) = e^{- \omega ^2 \sigma ^2 / 2}. \end{aligned}$$

It is clear that the filter is nonzero at $\omega = \omega _m + 2 \pi l$, $l \ne 0$, however, as can be appreciated from Fig. 7a, the attenuation at these frequencies is very strong. This makes the exponential reproduction very accurate as illustrated in Fig. 7b, c.

In the case of the Gaussian filter, we can easily obtain the $c_{m,n}$ coefficients of the exponentials to be reproduced since we have an analytical expression for its Fourier transform. When an analytic expression is unknown, we can still apply this approach since we only need knowledge of the transfer function of the acquisition device at frequencies $\omega = \omega _m$. The $c_{m,n}$ coefficients are then given by (22).

The approximate Strang-Fix framework is therefore very attractive since it allows us to use the theory discussed so far with any acquisition device.

### Perfect reconstruction of FRI signals

In the previous section, we have seen some properties of exponential reproducing kernels. We have also seen that if the sampling kernel satisfies the exponential reproducing property, we can obtain some samples of the Fourier transform of the input analogue signal from the measurements $\left( y_n\right) _{n=1}^{N}$ that result from the sampling process. We now show how this partial knowledge of the Fourier transform can be used to perfectly reconstruct some classes of band unlimited signals.

#### Perfect reconstruction of a stream of Diracs

We assume that the input signal is a stream of Diracs: $x(t) = \sum _{k=1}^K a_k \, \delta (t-t_k)$, and that the sampling kernel $\varphi (t)$ satisfies the exponential reproduction property for a choice of $\varvec{\alpha } = \left( \alpha _m \right) _{m=0}^P$ such that $\alpha _m = i \omega _m$, where $\omega _m \in \mathbb {R}$ for $m = 0, 1, \ldots , P$. We further impose the frequencies $\omega _m$ to be equispaced, that is $\omega _{m+1} - \omega _{m} = \lambda$, and to be symmetric, that is $\omega _m = -\omega _{P-m}$. We thus have $\omega _m = \omega _0 + \lambda m$ and $\omega _P = -\omega _0$.

Since $x(t)$ is a sum of Diracs, we have that the Fourier transform is given by a sum of exponentials:

24 $$\begin{aligned} \begin{aligned} \hat{x} (\omega )&= \int _{-\infty }^{+\infty } \sum _{k=1}^{K} a_k \, \delta (t-t_k) \, e^{-i \omega t} \hbox {d}t\\&= \sum _{k=1}^{K}a_k \, e^{-i \omega t_k}. \end{aligned} \end{aligned}$$

This is clearly a band unlimited signal. We now consider the sequence $s_m$ that is obtained by linearly combining samples $y_n$ with the coefficients $c_{m,n}$ from the exponential reproducing property (9). From (15), we have that $s_m = \hat{x} (-\omega _m / T)$ and therefore:

25 $$\begin{aligned} \begin{aligned} s_m&= \sum _{k=1}^K a_k \, e^{i \omega _m t_k / T}\\&= \sum _{k=1}^K \underbrace{a_k \, e^{i \omega _0 t_k / T}}_{b_k} \, \left( \underbrace{e^{i \lambda t_k / T}}_{u_k} \right) ^m\\&= \sum _{k=1}^K b_k \, u_k^m, \end{aligned} \end{aligned}$$

where $b_k {:=} a_k \, e^{i \omega _0 t_k / T}$ and $u_k {:=} e^{i \lambda t_k / T}$. Note that we have also applied the fact that the frequencies can be expressed as $\omega _m = \omega _0 + \lambda m$. The perfect recovery of the original stream of Diracs, that is, the estimation of the locations $t_k$ and the amplitudes $a_k$ of the $K$ Diracs, is now recast as the estimation of parameters $b_k$ and $u_k$ from the knowledge of values $s_m$. The problem of estimating the parameters of a sum of exponentials from a set of samples arises in a variety of fields and has been analysed for several years by the spectral estimation community (Pisarenko 1973; Paulraj et al. 1985; Schmidt 1986). One way to solve it is by realising that the sequence $s_m$ given as in (25) is the solution to the following linear homogeneous recurrence relation

26 $$\begin{aligned} h_K \, s_{m-K} + \cdots + h_1 \, s_{m-1} + s_m = 0. \end{aligned}$$

See section “Linear homogeneous recurrence relations with constant coefficients” of Appendix for a description of this type of homogeneous systems and their solutions. Note that coefficients $h_1, \ldots , h_K$ are unknown, but can be obtained from the following linear system of $K$ equations:

27 $$\begin{aligned} \begin{bmatrix} s_{K-1}&s_{K-2}&\ldots&s_{0} \\ s_{K}&s_{K-1}&\ldots&s_{1} \\ \vdots&\vdots&\ddots&\vdots \\ s_{2K-2}&s_{2K-3}&\ldots&s_{K-1} \end{bmatrix} \cdot \begin{bmatrix} h_1 \\ h_2 \\ \vdots \\ h_K \end{bmatrix} = - \begin{bmatrix} s_{K} \\ s_{K+1} \\ \vdots \\ s_{2K-1} \end{bmatrix}. \end{aligned}$$

It can be shown that, if the $K$ parameters $u_k$ in (25) are distinct, which is a direct consequence of the fact that all the delays $t_k$ are different, the Toeplitz matrix in the left-hand side of (27) is of rank $K$, and therefore, the solution is unique (see section “Rank deficiency of Toeplitz matrix” of Appendix for a proof on the rank of this matrix). As shown in section “Linear homogeneous recurrence relations with constant coefficients” of Appendix, the parameters $u_k$ are obtained from the roots of the polynomial $H(z) = h_K \, z^{-K} + \cdots + h_1 \, z^{-1}+1$. Once the parameters $u_k$ have been obtained, the amplitudes $b_k$ of the sum of exponentials can be directly retrieved from (25) by solving the associated least squares problem. From $u_k$ and $b_k$, we can then compute $t_k$ and $a_k$. The stream of Diracs is thus perfectly recovered. In the literature, this approach is known as Prony’s method or the annihilating filter method (Stoica and Moses 2005).

The system of equations (27) requires at least $2K$ consecutive values $s_m$. Recall that the sequence $s_m$ is obtained as follows $s_m = \sum _{n=1}^{N} c_{m,n}\,y_n$, with $m=0,1,\ldots ,P$, where $P+1$ is the number of exponentials reproduced by the sampling kernel. We thus have a lower bound on the number of exponentials that the sampling kernel has to reproduce: $P+1 \ge 2K$. The perfect reconstruction of a stream of Diracs is summarised in the following theorem.

##### **Theorem 1**

Consider a stream $x(t)$ of K Diracs: $x(t) = \sum _{k=1}^{K} a_k \, \delta (t-t_k)$, and a sampling kernel $\varphi (t)$ that can reproduce exponentials $e^{\,i(\omega _0+\lambda \,m)t}$, with $m=0,1,\ldots ,P$, and $P+1\ge 2K$. Then, the samples defined by $y_n = \left\langle x(t)\,,\,\varphi (t/T-n)\right\rangle$ are sufficient to characterise $x(t)$ uniquely.

**Fig. 8:**
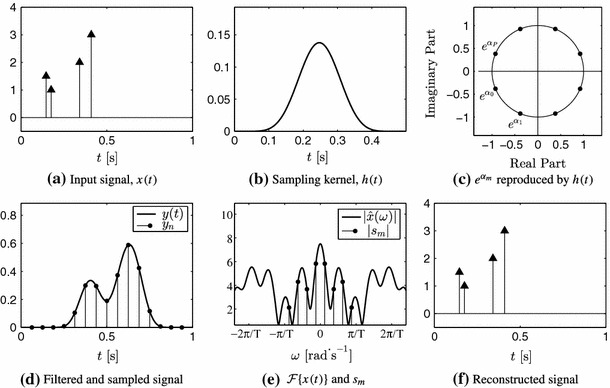
Sampling of a stream of Diracs and perfect reconstruction. $K=4$ Diracs sampled with an E-spline of order $P=7$ which corresponds to the critical sampling rate ($P+1 = 2K$). **a** is the continuous-time stream of Diracs, **b** the sampling kernel $h(t) = \varphi (-t/T)$ where $\varphi (t)$ is an E-spline of order $P=7$ that reproduces the exponentials illustrated in **c**. **d** is the continuous-time signal $y(t) = x(t) * h(t)$ and the corresponding discrete samples $y_n = y(t)|_{t = nT}$. In **e**, $|\hat{x}(\omega )|$ is obtained from (24) and $|s_m|$ from samples $y_n$ linearly combined with coefficients $c_{m,n}$. **f** is the reconstructed stream of Diracs from the sequence $s_m$. The original signal is perfectly reconstructed

Figure 8 illustrates the entire sampling process. Note that, since the sampling kernel is of compact support and the stream of Diracs is localised in time, there are only a small number of samples $y_n$ that are nonzero. From Fig. 8e, it is clear that the signal is not bandlimited. Furthermore, in the classical sampling setup, in order to sample a continuous-time signal at rate $T^{-1}$ Hz, an anti-aliasing filter that sets to zero $\hat{x}(\omega )$ for $|\omega | \ge \pi / T$ has to be applied before acquisition. The FRI framework does not impose this stringent condition since the sampling kernel is not necessarily equal to zero for all $|\omega | \ge \pi / T$.

#### Perfect reconstruction of a stream of decaying exponentials

Streams of Diracs are an idealisation of streams of pulses. Although this example may seem limited, the framework presented so far can be applied to other classes of functions that model a variety of signals. For instance, calcium concentration measurements obtained from two-photon imaging to track the activity of individual neurons can be modelled with a stream of decaying exponentials. In this model, the time delays correspond to the activation time of the tracked neuron, that is, the action potentials (AP).

**Fig. 9:**
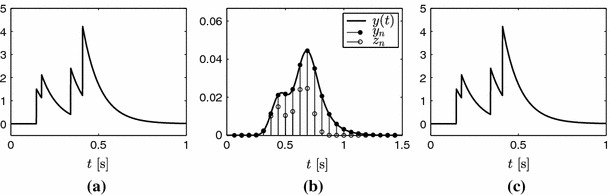
Sampling of a stream of decaying exponentials and perfect reconstruction. Since $x(t)$ is an infinite duration signal, samples $y_n$ are nonzero for $n \ge n_0$, for some $n_0$ that depends on the location of the first decaying exponential. However, if the number of decaying exponentials is finite, the number of nonzero samples $z_n = y_n - y_{n-1}\,e^{-\alpha T}$ is also finite since they are equivalent to sampling a stream of Diracs with a compact support kernel. **a** Input signal, $x(t)$, **b** filtered and sampled signal, **c** reconstructed signal

Let $x(t)$ be a stream of $K$ decaying exponentials, that is

28 $$\begin{aligned} x(t) = \sum _{k=1}^{K} a_k \, e^{-\alpha (t-t_k)}\, \varvec{1}_{t \ge t_k} = \sum _{k=1}^{K} a_k \, \rho _{\alpha }(t-t_k), \end{aligned}$$

where $\rho _{\alpha }(t) {:=} e^{-\alpha t} \, \varvec{1}_{t \ge 0}$. See Fig. 9a for an example of such signal. This is also an FRI signal since $x(t)$ is perfectly determined by a finite number of parameters: $\{(t_k, a_k)\}_{k=1}^{K}$. Let us assume that $x(t)$ is sampled with the acquisition device described in Sect. 3.2.1, that is, an exponential reproducing kernel $h(t)=\varphi (-t/T)$, followed by a sampling stage. We thus have that $\varphi (t)$ satisfies (9), and the resulting samples $y_n$ can be expressed as the inner product between $x(t)$ and $\varphi (t/T-n)$ as in (8).

Let us also assume that the reproduced exponentials $e^{i \omega _m t}$ can be expressed as $e^{i(\omega _0+\lambda m)t}$, with $m=0,1,\ldots ,P$. It can be shown that sampling the signal in (28) with $\varphi (-t/T)$ and computing the following finite differences

29 $$\begin{aligned} z_n = y_n - y_{n-1} \, e^{-\alpha T}, \end{aligned}$$

is equivalent to the sequence that would result from sampling the stream of Diracs $s(t) = \sum _{k=1}^{K} a_k \, \delta (t-t_k)$ with the following kernel

30 $$\begin{aligned} \psi (t) = \beta _{\alpha T} (-t) * \varphi (t) \end{aligned}$$

where $\beta _{\alpha T} (-t)$ is a zero order E-spline with parameter $\alpha T$ (Oñativia et al. 2013a). Note that $\alpha$ is the exponent in (28). We thus have that

31 $$\begin{aligned} z_n = \left\langle s(t)\,,\,\psi (t/T-n)\right\rangle . \end{aligned}$$

Since convolution preserves the exponential reproduction property, $\psi (t)$ reproduces the same exponentials as $\varphi (t)$. Thus, we can find the coefficients $d_{m,n}$ such that

32 $$\begin{aligned} \sum _{n \in \mathbb {Z}} d_{m,n} \, \psi (t-n) = e^{i \omega _m t}, \quad m = 0, 1, \ldots , P. \end{aligned}$$

We now have all the elements to perfectly reconstruct the stream of decaying exponentials $x(t)$ from samples $y_n$, that is, estimate the set of pairs of parameters $\{(t_k, a_k)\}_{k=1}^{K}$. By combining the sequence $z_n$ with coefficients $d_{m,n}$, we obtain exactly the same measurements $s_m$ as in (25):

33 $$\begin{aligned} s_m = \sum _{n=1}^{N} d_{m,n} \, z_n = \sum _{k=1}^{K} b_k \, u_k^m, \end{aligned}$$

where $b_k = a_k \, e^{i \omega _0 t_k / T}$ and $u_k = e^{i \lambda t_k / T}$. We can therefore apply Prony’s method to this sequence and obtain the parameters of interest. Figure 9 illustrates the perfect reconstruction of a stream of $K=4$ decaying exponentials.

### FRI signals with noise

The acquisition process inevitably introduces noise making the solutions described so far only ideal. Perturbations may arise in the analogue and digital domain. We model the noise of the acquisition process as a white Gaussian process that is added to the ideal samples. The noisy samples are therefore given by

34 $$\begin{aligned} \tilde{y}_n = y_n + \varepsilon _n, \end{aligned}$$

where $y_n$ are the ideal noiseless samples from (8) and $\varepsilon _n$ are i.i.d. Gaussian random variables with zero mean and variance $\sigma _{\varepsilon }^2$. In order to have a more robust reconstruction, we increase the number of samples $s_m$ by making the order $P$ larger than the critical rate $2K-1$.

The denoising strategies that can be applied to improve the performance of the reconstruction process come from the spectral analysis community, where the problem of finding sinusoids in noise has been extensively studied. There are two main approaches. The first, named Cadzow denoising algorithm, is an iterative procedure applied to the Toeplitz matrix constructed from samples $s_m$ as in (27). Let us denote by $\varvec{S}$ this matrix. By construction, this matrix is Toeplitz, and in the noiseless case, it is of rank $K$. The presence of noise makes this matrix be full rank. The Cadzow algorithm (Cadzow 1988) looks for the closest rank deficient matrix which is Toeplitz. At each step, we force matrix $\varvec{S}$ to be of rank $K$ by computing the singular value decomposition (SVD) and only keeping the $K$ largest singular values and setting the rest to zero. This new matrix is not Toeplitz anymore, we thus compute a new Toeplitz matrix by averaging the diagonal elements. This last matrix might not be rank deficient, and we can thus iterate again. The next step is to solve equation (27). This is done computing the total least squares solution that minimises $||\varvec{S} \varvec{h}||^2$ subject to $||\varvec{h}||^2=1$, where $\varvec{h}$ is an extended version of the vector in (27) and has length $K+1$. If this vector is normalised with respect to the first element, we have that the following $K$ elements correspond to the coefficients $h_k$ in (26). This approach has successfully been applied in the FRI setup in (Blu et al. 2008).

The second approach is based on subspace techniques for estimating generalised eigenvalues of matrix pencils (Hua and Sarkar 1990, 1991). Such approach has also been applied in the FRI framework (Maravić and Vetterli 2005). This method is based on the particular structure of the matrix $\varvec{S}$, which is Toeplitz and each element is given by a sum of exponentials. Let $\varvec{S}_0$ be the matrix constructed from $\varvec{S}$ by dropping the first row and $\varvec{S}_1$ the matrix constructed from $\varvec{S}$ by dropping the last row. It can be shown that in the matrix pencil $\varvec{S}_0 - \mu \varvec{S}_1$ the parameters $\left\{ u_k \right\} _{k=1}^K$ from (25) are rank reducing numbers, that is, the matrix $\varvec{S}_0 - \mu \varvec{S}_1$ has rank $K-1$ for $\mu = u_k$ and rank $K$ otherwise. The parameters $\left\{ u_k \right\} _{k=1}^K$ are thus given by the eigenvalues of the generalised eigenvalue problem $(\varvec{S}_0 - \mu \varvec{S}_1)\varvec{v} = 0$.

Further variations of these two fundamental approaches have been proposed recently. See for example Tan and Goyal (2008), Erdozain and Crespo (2011), Hirabayashi et al. (2013).

## Sampling streaming FRI signals

In the previous section, we have seen how to sample and reconstruct a set of $K$ Diracs. We now consider the case where we have a streaming signal:

35 $$\begin{aligned} x(t) = \sum _{k \in \mathbb {Z}} a_k \, \delta (t-t_k). \end{aligned}$$

If the stream is made of clearly separable bursts, we can apply the previously described strategy by assuming that each burst has a maximum number of spikes. However, when this separation cannot be made because of the presence of noise, or due to the nature of the signal itself, this strategy is not valid. The infinite stream presents an obvious constraint due the number of parameters that have to be recovered. We have seen that the order of the sampling kernel, $P$, and its support are directly related to the number of parameters to be estimated. However, we cannot increase $P$ indefinitely. In order to handle this type of signals, we thus consider a sequential and local approach (Oñativia et al. 2013b).

### Sliding window approach

We assume that $x(t)$ has a bounded local rate of innovation of $2K/\tau$, that is, for any time window of duration $\tau$ there are at most $K$ Diracs within the window. Since each Dirac has two degrees of freedom, location and amplitude, the rate of innovation is $2K/\tau$. We analyse sequentially the infinite stream with a sliding window that progresses in time by steps equal to the sampling interval $T$. Let the $i$-th window cover the following temporal interval

36 $$\begin{aligned} t \, \in \, ( n_i\,T, \, n_i\,T + \tau ], \end{aligned}$$

where $\tau = N \, T$ and $N$ is the number of samples that are processed for each position of the sliding window. The acquisition device is the same as in the previous section: the sampling kernel is given by $h(t)=\varphi (-t/T)$ and $y_n = \left\langle x(t)\,,\,\varphi (t/T-n)\right\rangle$. In order to have a causal filter $h(t)$, that is $h(t)=0$ for $t<0$, we impose the support of $\varphi (t)$ to be $t \, \in \, (-L,0]$, where $L=P+1$ if $\varphi (t)$ is an E-spline of order $P$. The support of $\varphi (t/T-n)$ is therefore $t \, \in \, ((n-L)T,nT]$. Consequently, a Dirac located at $t = t_k$ influences $L$ samples $y_n$. The indices corresponding to these samples are given by

37 $$\begin{aligned} \lceil {t_k/T}\rceil \le n < \lceil {t_k/T}\rceil + L. \end{aligned}$$

**Fig. 10:**
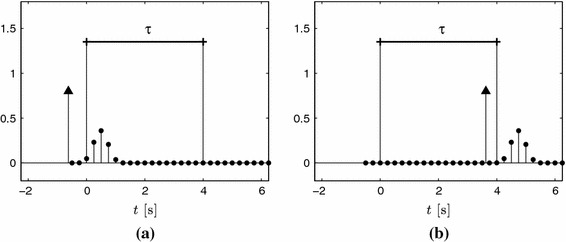
Border effects with the sliding window approach. In this example, $N=16$ and $T=1/4$. **a** A nearby Dirac located before the observation window $\tau$ influences samples $y_n$ of the window. **b** A Dirac inside the window but close to the *right* border generates nonzero samples outside the window

When we process the stream sequentially, there are border effects due to the fact that we only process $N$ samples at a time. Diracs located just before the sliding window influence samples within the window, and the Diracs inside the observation window which are close to the right border influence samples outside the window. These effects are illustrated in Fig. 10. However, if the sliding window is big enough, there are a good number of positions of the sliding window that will fully capture each individual Dirac and therefore lead to a good estimate of its amplitude and location. In the noiseless case, we can detect if we are in the presence of these border effects or if there is no border effect and therefore the reconstruction can be exact. Nonetheless, in the presence of noise, we cannot guarantee perfect reconstruction.

**Fig. 11:**
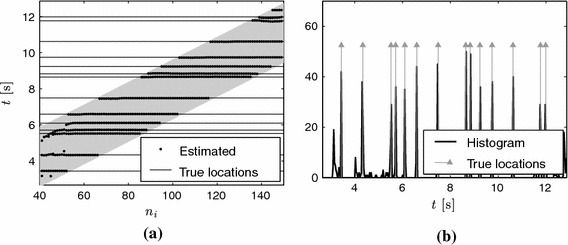
Noisy scenario with SNR = 15 dB, $N=50$ and $T=1/16$. The maximum rate of innovation of the streaming signal is $2K/\tau = 3.2 (K=5)$. **a** Plot of the sequentially estimated locations, the horizontal axis indicates the index of the sliding window and the vertical axis the location in time. **b** Histogram of the locations shown in **a**. Horizontally aligned *dots* in **a** lead to peaks in the histogram in **b**

For this reason, the sequential algorithm works in two steps: first, it estimates the locations for each position of the sliding window; second, it analyses the consistency of the retrieved locations among different windows. The $i$-th window processes samples $\left( \tilde{y}_n \right) _{n=n_i+1}^{n_i+N}$. Let $\lbrace \hat{t}_k^{(i)} \rbrace$ be the set of estimated locations within the $i$-th window. When the observation window is at position $t = n_i\,T$, we know that Diracs located at $t < (n_i-L)/T$ cannot have any influence on the current samples. We can therefore analyse the consistency of the locations up to $(n_i-L)/T$. Figure 11a shows the retrieved locations for different positions of the sliding window, where the horizontal axis corresponds to the window index, $n_i$, and the vertical axis to the locations in time, that is, for a given window index, each dot corresponds to an estimate of the set $\lbrace \hat{t}_k^{(i)} \rbrace$. Consistent locations among different windows appear as horizontally aligned dots. The shaded area represents the evolution in time of the observation window: for a given index $n_i$, the vertical cross section of the shaded area represents the time interval $\tau$ that is *seen* by this window. This consistency can be analysed by building a histogram of all the estimated locations up to a given time. This is illustrated in Fig. 11b. The Diracs are then estimated from the peaks of this histogram.

## Application to neuroscience

To understand how neurons process information, neuroscientists need accurate information about the firing of action potentials (APs of spikes) by individual neurons. We thus need techniques that allow to monitor large areas of the brain with a spatial resolution that distinguishes single neurons and with a temporal resolution that resolves APs. Of the currently available techniques, only multiphoton calcium imaging (Denk et al. 1990, 1994; Svoboda et al. 1999; Stosiek et al. 2003) and multielectrode array electrophysiology (Csicsvari et al. 2003; Blanche et al. 2005; Du et al. 2009) offer this capability. Of these, only multiphoton calcium imaging currently allows precise three-dimensional localisation of each individual monitored neuron within the region of tissue studied, in the intact brain. Populations of neurons are simultaneously labelled with a fluorescent indicator, acetoxy-methyl (AM) ester calcium dyes (Stosiek et al. 2003). This allows simultaneous monitoring of action potential-induced calcium signals in a plane (Ohki et al. 2005) or volume (Göbel and Helmchen 2007) of tissue. The calcium concentration is measured with a laser-scanning two-photon imaging system.

For a given region of interest (ROI) where a neuron is located, the calcium concentration is obtained by averaging the value of the pixels of the ROI for each frame. The result is a one-dimensional fluorescence sequence. We assume that when a neuron is activated, the calcium concentration jumps instantaneously, and each jump has the same amplitude $A$. The concentration then decays exponentially, with time constant $\tau$, to a baseline concentration. The one-dimensional fluorescence signal can therefore be characterised by convolving the spike train with a decaying exponential and adding noise:

38 $$\begin{aligned} \begin{aligned} c(t)&= A \, \sum _{k} e^{-(t-t_k) / \tau } \, \varvec{1}_{t \ge t_k} + \varepsilon _t\\&= A \, \sum _{k} \delta (t-t_k) * e^{-t / \tau } \, \varvec{1}_{t \ge 0} + \varepsilon _t, \end{aligned} \end{aligned}$$

where the index $k$ represents different spikes and the different $t_k$ their occurrence times. Hence, the goal of spike detection algorithms is to obtain the values $t_k$.

A number of methods have previously been used to detect spike trains from calcium imaging data, including thresholding the first derivative of the calcium signal (Smetters et al. 1999), and the application of template-matching algorithms based on either fixed exponential (Kerr et al. 2005, 2007; Greenberg et al. 2008) or data-derived (Schultz et al. 2009; Ozden et al. 2008) templates. Machine learning techniques (Sasaki et al. 2008) and probabilistic methods based on sequential Monte Carlo framework (Vogelstein et al. 2009) or fast deconvolution (Vogelstein et al. 2010) have also been proposed. Some broadly used methods such as template matching or derivative-thresholding have the disadvantage that they do not deal well with multiple events occurring within a time period comparable to the sampling interval. Our spike detection algorithm is based on connecting the calcium transient estimation problem to the theory of FRI signals. The calcium concentration model in (38) is clearly a FRI signal, we can thus apply the techniques presented in the previous sections.

### Spike inference algorithm

The spike inference algorithm is based on applying the sliding window approach presented in Sect. 4.1 combined with the reconstruction of streams of decaying exponentials presented in Sect. 3.2.2. One major issue of the framework presented so far is that we have assumed the number $K$ of spikes within a time window to be known a priori. In practice, this is a value that has to be estimated.

In the noiseless case, the number of spikes can be recovered from the rank of the Toeplitz matrix constructed from samples $s_m$:

39 $$\begin{aligned} \varvec{S} = \begin{bmatrix} s_{\lceil {P/2}\rceil }&\ldots&s_{0} \\ \vdots&\ddots&\vdots \\ s_{P}&\ldots&s_{P-\lceil {P/2}\rceil } \end{bmatrix}. \end{aligned}$$

In the noisy case, matrix $\varvec{S}$ becomes full rank. An estimate of $K$ can still be obtained by thresholding the normalised singular values of $\varvec{S}$. Let $\mu _1 \ge \mu _2 \ge \ldots \mu _{\lfloor {P/2}\rfloor + 1}$ be the singular values of $\varvec{S}$ sorted in decreasing order. We can estimate $K$ as the number of singular values that satisfy $\mu _i / \mu _1 \ge \mu _0$. Where $0<\mu _0<1$ is adjusted depending on the level of noise. This approach tends to overestimate $K$. Moreover, we never detect the $K=0$ case since when noise is present we always have $\mu _1 \ne 1$.

**Fig. 12:**
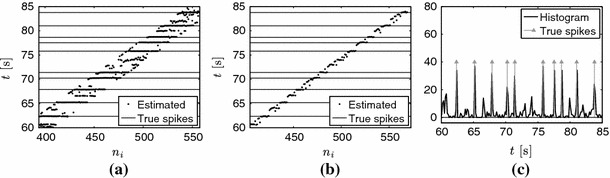
Double consistency spike search with real data. **a** and **b** show the detected locations with *dots* and the original spikes with *horizontal lines* for two different window sizes. In **a**, the algorithm runs estimating the number of spikes within the sliding window. In **b**, the algorithm runs assuming a fixed number of spikes equal to one for each position of the sliding window. **c** shows the joint histogram of the detected locations

**Fig. 13:**
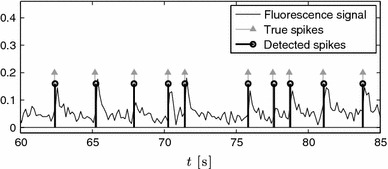
Fluorescence signal and detected spikes using the double consistency approach. The spikes are detected from the peaks of the histogram in Fig. 12c

To overcome these inaccuracies, we make the algorithm more robust by applying a double consistency approach. We run the sliding window approach presented in Sect. 4.1 twice. First, with a sufficiently big window where we estimate $K$ from the singular values of $\varvec{S}$. Second, with a smaller window where we assume that we only capture one spike and therefore we always set $K=1$. We then build a joint histogram out of all the locations retrieved from both approaches and estimate the spikes from the peaks of the histogram. This approach is illustrated in Figs. 12 and 13 with real data.

This technique is fast and robust in high noise and low temporal resolution scenarios. It is able to achieve a detection rate of 84 % of electrically confirmed AP with real data (Oñativia et al. 2013a), outperforming other state of the art real-time approaches. Due to its low complexity, tens of streams can be processed in parallel with a commercial off-the-shelf computer.

## Conclusions

We have presented a framework to sample and reconstruct signals with finite rate of innovation. We have shown that it is possible to sample and perfectly reconstruct streams of Diracs, and more importantly, streams of decaying exponentials. The latter offer a perfect fit for calcium transients induced by the spiking activity of neurons. The presented approach is sequential, and the reconstruction is local. These two features make the overall algorithm resilient to noise and have low complexity offering real-time capabilities.

The theoretical framework, where perfect reconstruction can be achieved, is also extended to the more realistic case where we do not have full control over the sampling kernel. In this case, perfect reconstruction cannot be guaranteed, but we can still reconstruct the underlying analogue signal with high precision if the sampling kernel can reproduce exponentials approximately.

## Appendix

### Linear homogeneous recurrence relations with constant coefficients

Let $L_K \left[ \cdot \right]$ be the linear operator with constant coefficients that establishes the following recurrence relation of order up to $K$ when applied to a sequence $y_n$:

40 $$\begin{aligned} L_K \left[ y_n \right] = h_K \, y_{n-K} + \cdots + h_1 \, y_{n-1} + y_n. \end{aligned}$$

The corresponding homogeneous system is given by

41 $$\begin{aligned} L_K \left[ y_n \right] = 0. \end{aligned}$$

This is the discrete-time version of a homogeneous linear differential equation given by

42 $$\begin{aligned} h_K \, \frac{d^{K} y(t)}{t^K} + \cdots + h_1 \, \frac{\hbox {d}y(t)}{\hbox {d}t} + y(t) = 0. \end{aligned}$$

Both, the linear homogeneous differential equation (42) and the linear homogeneous recurrence relation (41) have equivalent solutions. In the continuous-time case, the functions that satisfy the homogeneous equation have the form of exponential functions. Similarly, the solution to the discrete-time version has the form of exponential sequences. The solution to (41) is not unique, but all the solutions have the form $z^n$, where $z \in \mathbb {C}$. Thus, to solve (41) we set $y_n=z^n$, leading to

43 $$\begin{aligned} h_K \, z^{-K}\,z^n + \cdots + h_1 \, z^{-1}\,z^n + z^n = 0. \end{aligned}$$

Division by $z^n$ gives the $K$th order polynomial

44 $$\begin{aligned} H(z) = h_K \,z^{-K} + \cdots + h_1 \, z^{-1} + 1. \end{aligned}$$

$H(z)$ is the characteristic polynomial of the homogeneous system. The roots of $H(z)$, that is, the values $z_1, z_2, \ldots , z_K$ that satisfy $H(z_k) = 0$, determine the solution to (41). We have that $L_K \left[ z_k^n \right] = 0$. If the $K$ roots are distinct, the solution to the homogeneous recurrence relation is given by any linear combination of the sequences constructed from the different roots:

45 $$\begin{aligned} y_n = \sum _{k=1}^K a_k \, z_k^n \Longleftrightarrow L_K \left[ y_n \right] = 0, \end{aligned}$$

since $$L_K \left[ z_k^n \right] \!=\! 0$$ and $$L_K \!\left[ \sum _{k=1}^K a_k \, z_k^n \right] \!=\! \sum _{k=1}^K a_k \, L_K \left[ z_k^n \right] $$.

### Rank deficiency of Toeplitz matrix

Let $\varvec{S}$ be the following $(P-M+1)\times (M+1)$ Toeplitz matrix:

46 $$\begin{aligned} \varvec{S} = \begin{bmatrix} s_{M}&s_{M-1}&\ldots&s_{0} \\ s_{M+1}&s_{M}&\ldots&s_{1} \\ \vdots&\vdots&\ddots&\vdots \\ s_{P}&s_{P-1}&\ldots&s_{P-M} \end{bmatrix} , \end{aligned}$$

where $(P-M+1) \ge K$, $(M+1) \ge K$ and each element of the matrix is given by $s_m = \sum _{k=1}^{K} b_k \, u_k^m$, with all $b_k$ nonzero and all $u_k$ distinct. The matrix $\varvec{S}$ can be decomposed as follows:

47 $$\begin{aligned} \varvec{S} \!\!=\!\! \underbrace{ \begin{bmatrix} 1&\ldots&1 \\ u_1&\ldots&u_K \\ \vdots&\ddots&\vdots \\ u_1^{P-M}&\ldots&u_K^{P-M} \\ \end{bmatrix} }_{\varvec{B}} \, \underbrace{ \begin{bmatrix} b_1&\ldots&0 \\ \vdots&\ddots&\vdots \\ 0&\ldots&b_K \end{bmatrix} }_{\varvec{A}} \, \underbrace{ \begin{bmatrix} u_1^{M}&u_1^{M-1}&\ldots&1 \\ \vdots&\vdots&\ddots&\vdots \\ u_K^{M}&u_K^{M-1}&\ldots&1 \\ \end{bmatrix} }_{\varvec{C}} . \end{aligned}$$

Since $\varvec{B}$ and $\varvec{C}$ are Vandermonde matrices with distinct elements, both are of rank $K$. Therefore, if elements $b_1,b_2,\ldots ,b_K$ are nonzero, matrix $\varvec{S}$ has rank $K$.

## Footnotes

1 For appropriate functions $f$, the Poisson summation formula is given by: $\sum _{n=-\infty }^{+\infty } f (t - nT) = \frac{1}{T} \sum _{k=-\infty }^{+\infty } \hat{f} \left( \tfrac{2 \pi k}{T}\right) \, e^{i 2\pi k t / T}$.
